# Mechanism of histone deacetylases in cardiac hypertrophy and its therapeutic inhibitors

**DOI:** 10.3389/fcvm.2022.931475

**Published:** 2022-07-26

**Authors:** Yu Han, Jiali Nie, Dao Wen Wang, Li Ni

**Affiliations:** ^1^Division of Cardiology, Department of Internal Medicine, Tongji Hospital, Tongji Medical College, Huazhong University of Science and Technology, Wuhan, China; ^2^Hubei Key Laboratory of Genetics and Molecular Mechanisms of Cardiological Disorders, Wuhan, China

**Keywords:** cardiac hypertrophy, epigenetics, histone deacetylase, gene regulation, small molecule inhibitors

## Abstract

Cardiac hypertrophy is a key process in cardiac remodeling development, leading to ventricle enlargement and heart failure. Recently, studies show the complicated relation between cardiac hypertrophy and epigenetic modification. Post-translational modification of histone is an essential part of epigenetic modification, which is relevant to multiple cardiac diseases, especially in cardiac hypertrophy. There is a group of enzymes related in the balance of histone acetylation/deacetylation, which is defined as histone acetyltransferase (HAT) and histone deacetylase (HDAC). In this review, we introduce an important enzyme family HDAC, a key regulator in histone deacetylation. In cardiac hypertrophy HDAC I downregulates the anti-hypertrophy gene expression, including Kruppel-like factor 4 (Klf4) and inositol-5 phosphatase f (Inpp5f), and promote the development of cardiac hypertrophy. On the contrary, HDAC II binds to myocyte-specific enhancer factor 2 (MEF2), inhibit the assemble ability to HAT and protect against cardiac hypertrophy. Under adverse stimuli such as pressure overload and calcineurin stimulation, the HDAC II transfer to cytoplasm, and MEF2 can bind to nuclear factor of activated T cells (NFAT) or GATA binding protein 4 (GATA4), mediating inappropriate gene expression. HDAC III, also known as SIRTs, can interact not only to transcription factors, but also exist interaction mechanisms to other HDACs, such as HDAC IIa. We also present the latest progress of HDAC inhibitors (HDACi), as a potential treatment target in cardiac hypertrophy.

## Introduction

Myocardial hypertrophy is a key process of cardiac remodeling, which often occurs after high load in cardiac or myocardial infarction (1). Cardiac hypertrophy is generally considered to be a compensatory effect that reduces oxygen consumption, normalizes the ventricle systolic pressure, and improves the ejection function in a short term. However, long-term stress, such as hypertension, leads to adverse stimulation and ultimately irreversible pathological cardiac remodeling. The abnormal enlargement of myocardium and thickening of ventricle wall leading to cardiac dysfunction and fibrosis (2, 3). A variety of biological regulation processes are involved in cardiac hypertrophy and remodeling. In addition to the known mechanisms of cardiac hypertrophy, such as MAPK pathway, PI3K-AKT pathway, Calcineurin-NFATc and other signaling transduction pathways (4), histone acetylation and deacetylation mediated by epigenetic modification have attracted increasing attention from researchers. Histone acetylation and deacetylation play a crucial role in regulating gene expression and leading to hypertrophy under stress.

In the acetylation and deacetylation process, there is a group of molecules family- the HDACs family, which includes 4 major classes (HDAC I, HDAC IIa, HDAC IIb, and HDAC III), each with distinct expression patterns (5). HDAC I (HDAC 1, 2, 3, and 8) has deacetylation catalysis (6). HDAC II includes subclasses HDAC IIa (HDAC 4, 5, 7, and 9) and HDAC IIb (HDAC 6 and 10) (7). HDAC III—also known as sirtuins (SIRT1-7) (8). Recent studies indicate that HDAC I and IIa play important but opposite role in cardiology research, especially in cardiac hypertrophy. Although most HDAC have conserved catalytic domains, their expression and function appear to conform to a cell-specific patterns. As for class III HDACs, Sirt1 transgenic overexpression in mice shows effect of preventing cardiac apoptosis and hypertrophy induced by oxidative stress and aging (9). There is increasing evidence of the importance of different classes of HDACs in cardiac diseases including cardiac hypertrophy and heart failure. These effects can be reversed with the using of HDAC inhibitors (HDACi), suggesting HDACs may be novel therapeutic targets for preventing cardiac hypertrophy development.

In this review, we introduce the differences among the HDAC subclasses, and elucidate the pathophysiological function of HDACs in cardiac diseases. We will highlight the physiological regulation of HDACs in cardiac hypertrophy. Finally, we introduce the therapeutic value of HDACs as targets in regulating epigenetic modification against cardiac hypertrophy.

## Epigenetic regulations and histone acetylation

Epigenetic modification and non-coding RNA (ncRNAs) are major participants in epigenetic regulations (10). Epigenetic modifications can be roughly divided into following forms: methylation of cytosine residues on DNA (DNA methylation), post-translational modification of histones (proteins in which DNA is entangled in nucleosomes), and regulation of ncRNAs (11). These can not only directly affect cardiac disease gene expression at the post-transcriptional level (12), but also interact with other epigenetic regulations (crosstalk mechanisms) (13).

Among the modifications of histones, acetylation and methylation are the most studied, and other modifications, such as phosphorylation, have also been extensively researched in a variety of disease processes (9, 14). Meanwhile, interactions between non-coding RNA or DNA methylation modifications or histone modifications have also been shown to influence epigenetic regulatory processes (15), especially during cardiac hypertrophy (13).

There is an HDAC inhibitor- SAHA/vorinostat (Zolinza) with the FDA approval for cutaneous T cell lymphoma treatment as early as 2006 (16), indicating high value of modulating histone acetylation and deacetylation as a potential therapeutic target. Today, many studies show HDAC have highly recognized therapeutic value in different diseases, including cardiac hypertrophy (17). In this article, we will focus on histone modification, especially histone acetylation and deacetylation.

## Histone acetylation and deacetylation

Histone acetylation plays an important role in histone modifications, and affects cardiac diseases (18). Histone acetyltransferase (HAT) and histone deacetylase (HDAC) modulate histone acetylation/deacetylation dynamic balance. The disruption of this balance involves rearrangement of gene expression in the embryo, leading to cardiac hypertrophy (7, 19).

**FIGURE 1:**
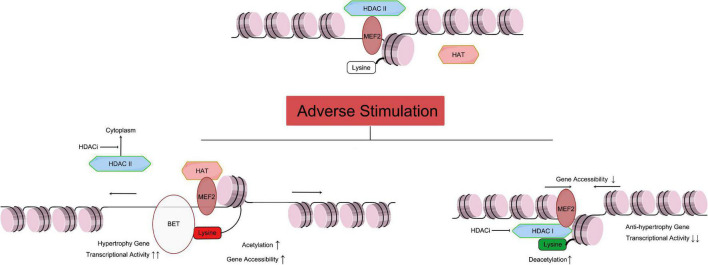
Relationship of HAT and HDAC mediated histone acetylation/deacetylation leading to cardiac hypertrophy. As mentioned above, the Class IIa HDACs are not capable of deacetylating histone residues due to a within the catalytic domain mutation. Therefore, HDAC IIa represses gene transcription by binding with MEF2, recruiting other transcriptional repressors and epigenetic regulators to DNA promoter regions, and maintaining the acetylation level of histone (Figure 1 top part). Under adverse stimulation such as pressure overload, HDAC IIa isolate from MEF2 and transport to cytoplasm, while MEF2 recruiting HAT and catalyze histone lysine residue, and regulate transcription activity. Meanwhile, BET family recognize the acetylation of histone, and bind to related gene promoter region, and promote cardiac hypertrophy (Figure 1 left bottom part). Meanwhile, Class I and IIb HDACs catalyze the removal of acetyl groups from key lysine residues within histone. Histone deacetylation induces chromatin condensation, which represses gene transcription by making gene promoter and enhancer regions less accessible to transcription. Overexpression of HDAC I reduces acetylation of lysine in histone (such as H3K27ac), which will reduce anti-hypertrophy gene transcriptional activity, leading to cardiac hypertrophy. HDACi can inhibit HDAC I catalyze activity, and stop the transport of HDAC II from nuclear to cytoplasm, and protect the heart. MEF, myocyte-specific enhancer; HAT, histone acetyltransferase; HDAC, histone deacetyltransferase; HDACi, HDAC inhibitor. [By Figdraw (www.figdraw.com)].

Histone acetylation occurs in epigenomic modification and is highly related to two families of enzyme that act contrarily: HATs and HDACs (20–22). The lysine residues are the working site of histone acetylation, promoting chromatin relaxation and activation of transcription process. In contrast, hypoacetylation of histone leads to gene expression inhibition caused by chromatin concentration (Figure 1). There are two main types of acetyl coenzyme. The nuclear type A, includes N-acetyltransferase 6 (NAT6), MYST family (MOZ, YBF2, SAS2, and TIP60), and CREB-binding protein (CBP)/p300, and the cytoplasmic type B (23). Researches suggest transcription of p300 is controlled by myocyte-specific enhancer factor 2 (MEF2) and GATA4 (zinc-finger transcription factor, indicating cardiac hypertrophy, and increasing DNA accessibility), and this transcript control process is critical for cardiac development (24, 25) and heart failure (26–28).

HDACs catalyze histone acetylated lysine residues of ε-amino groups (29). Histone terminal deacetylation leads to chromatin concentration, which structurally inhibits DNA accessibility, and leads to transcription inhibition (30). So far, there are 18 species of HDACs have been defined (Table 1). HDAC I and II require Zn^2+^ for enzyme activation. Differently, HDAC III-also known as sirtuins (SIRT1-7), whose enzyme activation relies more on nicotinamide adenine dinucleotide (NAD +) (5). In addition, HDACs contribute to not only histone, but also non-histone proteins, such as molecular chaperones, signaling molecules, and so on. In this way, HDACs affect the binding ability of histone and non-histone protein that regulates transcription and multiple biological processes (31, 32).

**TABLE 1:** Different classes of HDAC and a brief introduction of their basic characteristics.

Class	HDAC family	Localization	Major target molecular	Function to cardiac hypertrophy	Other effects	References
I	HDAC1	Nucleus	Mef2C	Promote	Cell proliferation, differentiation, development, cancer	(136)
	HDAC2	Nucleus	Inpp5f	Promote	Cell proliferation, development, synaptic plasticity, differentiation	(6, 51)
	HDAC3	Nucleus/Cytoplasm	Mef2C	Inhibit	Cell proliferation, development	(54)
	HDAC8	Nucleus	p38	Promote	Smooth muscle differentiation and contractility	(137)
IIa	HDAC4	Nucleus/Cytoplasm	Mef2C	Inhibit	Bone development, neuron development	(53)
	HDAC5	Nucleus/Cytoplasm	Mef2C	Inhibit	Bone development, axonal regeneration	(57)
	HDAC7	Nucleus/Cytoplasm	c-Myc	Promote	Vascular development, immunomodulation	(138)
	HDAC9	Nucleus/Cytoplasm	Mef2C	Inhibit	Neuron development, synaptic plasticity, immunomodulation	(139)
IIb	HDAC6	Cytoplasm	α-tubulin	Promote	Cytoskeletal dynamics, cell motility, aggresome formation, autophagy	(140)
	HDAC10	Nucleus/Cytoplasm	pRb	−	Cell cycle, immunomodulation, cancer	
III	SIRT1	Nucleus	PGC-1α	Inhibit	Cell survival, aging, energy metabolism, inflammation	(78)
	SIRT2	Nucleus	LKB1	Inhibit	Microtubule stability	(85)
	SIRT3	Cytoplasm	FoxO3a	Inhibit	Energy metabolism	(80)
	SIRT4	Cytoplasm	Sirt3	Promote	Energy metabolism	(141)
	SIRT5	Cytoplasm	ECHA	Inhibit	Urea cycle, apoptosis, energy metabolism	(142)
	SIRT6	Nucleus	NFATc4	Inhibit	Telomeric DNA redulation	(86)
	SIRT7	Nucleus	Histone 3	−	Apoptosis	
IV	HDAC11	Nucleus/Cytoplasm		Promote	Immunomodulation, energy metabolism	(143, 144)

ECHA, enoyl-coenzyme A hydratase; FoxO3, Forkhead box O3; Inpp5f, inositol-5 phosphatase f; NFATc4, nuclear factor of activated T-cells; PGC-1α, Peroxisome proliferator-activated receptor gamma coactivator-1 alpha.

## Histone acetyltransferase activates cardiac hypertrophy

Histone acetyltransferase inhibits the chromatin concentration, promotes DNA accessibility for transcription factors (33). As a common HAT, p300 plays an important role in cardiac hypertrophy. Its active state is usually binding to a co-activator CBP, forming a complex CBP/p300 (34). HAT activity increased during cardiac hypertrophy, and p300 overexpression in cardiomyocytes increased cardiac remodeling in adult mice models (27).

The HAT complex on one hand cooperates with transcription factors such as MEF2C, GATA4, and HIF1, and binds to transcriptional regulation elements such as promoters or enhancers to participate in cardiac hypertrophy. On the other hand, the HAT complex regulates the GATA4 activation through its catalytic acetylation, improving its binding ability with DNA (35, 36). Since CBP/p300 activates enhancers through acetylation of histone H3 lysine 27 residue (H3K27ac), the HAT complex is used to target regions of activity enhancer associated with cardiac physiological processes (37).

In animal model, left ventricular hypertrophy, dilation, and dysfunction occurred when CBP/p300 was overexpressed in cardiomyocytes (27). P300 overexpression in mouse cardiomyocytes stimulates the expression of the GATA4-dependent genes related to cardiac hypertrophy, including natriuretic peptide precursor A(*Nppa*, mediating the translation of ANP), prepro-endothelin-1(ET-1), and β-myosin heavy chain (*Myh7*) (38). In contrast, curcumin, a p300-specific HAT inhibitor, reduces the acetylation of H3 and H4 and leads to increased DNA accessibility to GATA4 in phenylephrine induced hypertrophy rat models (39). Therefore, inhibition of HAT, or increase deacetylation may be a potential direction in the treatment of cardiac hypertrophy.

## An introduction of histone deacetylase and their functional subgroups in cardiac hypertrophy

In general, HDACs remove the acetyl group from the histones which constitute the nucleosome. After catalyzed reactions, low acetylation of histones results in reduced space between nucleosomes and DNA, changes in conformation, triggers reduced accessibility of DNA to transcription factors, and leads to transcriptional inhibition (Figure 1). This catalytic process requires zinc ions (Zn^2+^) as cofactors. At the same time, HDACi can replace Zn^2+^, causing enzyme dysfunction (40).

The different nuclear/cytoplasm localization affects the function of HDACs. HDACs need to perform their functions in the nucleus, where their substrates locate (Table 1, primary target molecule). Nuclear localization of HDACs is achieved by nuclear localization signals (NLS) or co-localization with other proteins. As shown in Table 1, most HDACs contain an NLS, which is the key of nucleus locate, but some HDACs can be cytoplasmic. For example, most HDAC I are localized in the nucleus (Table 1, localization) due to the lack of a nuclear export signal (NES), and obtain nuclear regulatory mechanism through MEF2C/NFAT signaling pathway (41). HDAC1 and HDAC2 are mainly located in the nucleus. However, HDAC3 can also localize to the cytoplasm and possess not only nuclear input signals but also NES, which gives HDAC3 the ability to shuttle through the nuclear membrane and related function (42).

For HDAC II, HDAC 4, 5, and 7 are able to interacts with calcium/calmodulin dependent protein kinase (CAMK), and shuttle between cytoplasm and nucleus in muscle cells (42–44). Therefore, the CAMK-HDAC II/MEF2 pathway may play an important role in cardiac hypertrophy (45).

HDAC III, also known as sirtuins (SIRTs). Unlike HDAC I and II, the enzyme activity of SIRTs depends on the presence of nicotinamide adenine dinucleotide (NAD +) (46). Sirtuin is generally considered a protective agent against cardiac hypertrophy. However, different levels of SIRT1 overexpression seem to play a dual role in promoting or inhibiting the development of cardiac hypertrophy (20). Meanwhile, SIRT1 and 3 interact with nuclear and mitochondrial proteins (47), to mediate energy metabolism and ATP synthesis, which may also be a critical step leading to cardiac hypertrophy.

## HDAC I promotes cardiac hypertrophy

HDAC I mainly locates in the nucleus, including HDAC1, 2, 3, and 8 (48). In 2006, a study (6) showed that inhibition of HDAC I could control or even reduce the development of cardiac hypertrophy and reverse cardiac remodeling. With the further research, the mechanism of HDAC I regulating cardiac hypertrophy has been gradually revealed.

For instance, there has been evidence that HDAC1 and HDAC2 inhibit cardioprotective and anti-hypertrophic genes (49, 50) (Figure 1, lower left). In addition, *Hdac2* knockout mice were resistant to undesirable stimuli that induce cardiac hypertrophy (51), such as pressure overload and calcineurin stimulation. This activity associated with HDAC deacetylation of histones and down-regulation of expression of anti-hypertrophy genes, including Kruppel-like factor 4 (*Klf4*) and inositol-5 phosphatase f(*Inpp5f*). In this way, serum response-myocardin (52) and AKT-GSK3β (Glycogen synthase kinase 3β) pathway activity (51) are inhibited, both of which contribute to cardiac hypertrophy. Furthermore, in heart-specific HDAC3 knockout mice, HDAC3 cooperated with SMRT/n-CoR resulted in reduced histone acetylation near MEF2 (53), leading to abnormal energy metabolism and cardiac hypertrophy (54). Furthermore, sodium valproate (VPA), an inhibitor of HDACs, can reduce the process of hypertension, cardiac hypertrophy, and cardiac remodeling in rats with nephrovascular hypertension induced by two-kidney-two-clip (2K2C) (43). Moreover, the expression of HDAC8, HDAC2, TGF-β1, and connective tissue growth factor (CTGF) decreased in the 2K2C model with VPA intervention, suggesting that VPA has anti-hypertension and anti-hypertrophy effect (43).

## Regulation of HDACII in cardiac hypertrophy

HDAC II, including HDAC IIa (HDAC 4, 5, 7, and 9) and IIb (HDAC 6, 10), plays a role in nucleus and cytoplasm. In the two subclasses, HDAC IIa process the mutation of catalytic domain, hence the lack of deacetylase activity (55). And HDAC IIa N-terminal extension interacts with transcription factors in nucleus, such as MEF2 or heterochromatin protein 1 (HP1) (56, 57). HDAC IIb is different. For instance, HDAC6 is recognized containing two copies of catalytic domain, while the HDAC10 has one catalytic domain and a leucine-rich C-terminal domain, which links it to the cytoplasm (58).

HDAC IIa is generally considered to be an anti-hypertrophic molecule, whose function depends on binding and subsequently inhibition of MEF2 (56). MEFs is a family of myocyte-specific enhancers responsible for transcriptional regulation of cardiomyocyte development (59). MEFs promote the transcription regulation of cardiac hypertrophy in pathological condition, including sustained β-adrenergic receptor stimulation, angiotensin II (Ang II) infusion and pressure overload (60, 61). HDAC IIa usually combines with MEF2C. When cardiomyocytes are stimulated by pathological stress, HDAC transport out of the nucleus, and MEF2C recruits p300 into chromatin in the absence of HDAC II, increasing transcription of hypertrophy related genes (Figure 1, lower left). Two other post-translational modifications-phosphorylation and oxidation of the HDAC II, regulate the binding activity of HDAC II-MEF2C.

Calcium/calmodulin dependent protein kinase 2 (CaMKII) phosphorylates n-terminal serine residues on HDAC II in the presence of hypertrophic induction simulations. This process leads to the binding of a chaperone family of 14-3-3 to HDAC II, which results in the separation and transport of MEF2C to the cytoplasm (62, 63). Another study showed that HDAC4 has the same ability of translocation during cardiac hypertrophy due to the accumulation of reactive oxygen species (ROS). ROS oxidation of cysteine residues (64) suggests the interaction between HDAC II and cardiac hypertrophy.

### HDAC IIa interacts with other transcription factors in cardiac hypertrophy

Recent studies show that HDAC IIa (HDAC 4, 5, 7, and 9) located in the nucleus has an anti-hypertrophy effect (7, 65). Notably, HDAC appears to modulate specific pathways, such as protein kinase D (PKD) and CaMKIIδB, without affecting other pathways stimulated by β-adrenergic agonists (7).

As previously mentioned, HDAC IIa-MEF2 is involved in the process of inhibiting fetal gene transcription and inhibiting hypertrophy. HDAC4/SUV39H1 forms a repressive complex, maintaining the MEF2 nearby H3 methylation, and ANP, BNP expression decreased, suggesting a heart protection effect (66). As SUV39H1 is a nuclear histone methyltransferase, this interaction shows a crosstalk mechanism between histone deacetylation and histone methylation (67). In response to adverse stimulation, CaMKIIδB reduced HDAC4 phosphorylation and transport from the nucleus to the cytoplasm, thereby inducing increased levels of nuclear HDAC4, resulting in dissociation of the complex HDAC4/SUV39H1, demethylation of H3K9, transcriptional activation of Mef2, and ultimately cardiac hypertrophy (67).

In addition, cardiac hypertrophy-related GATA4-dependent genes expression, including *Nppa and Myh7*, also enhance the reaction to the adverse stimulation. Meanwhile, *Nppa* and *Myh7* gene expression were positively correlated with the acetylation level of histone 3 (68). The results suggested that HDAC IIa could inhibit cardiac hypertrophy. The knockout of *Hdac5* or *Hdac9* in cardiomyocytes increased the sensitivity of mice to adverse stimuli for cardiac hypertrophy, leading to cardiac hypertrophy and cardiomyopathy (7, 68). Mice with single knockout *Hdac5* or *Hdac9* are prone to chronic hypertrophy in response to adverse stimulation of hypertrophy (7, 68).

There are other interaction mechanisms between HDAC and other HDAC molecules. HDAC5, together with HDAC1, can form a regulatory compound, which accumulates p300 to the promoter region of *Ncx1* (sodium calcium exchanger) gene and up-regulates the transcription of Ncx1 (69). Meanwhile, Nkx2.5, as sodium potassium exchanger, also deacetylates increasingly. As a result, the HDAC5/1 complex induces a calcium overload process leading to cardiac hypertrophy (69). These results suggest complex regulating mechanisms of HDAC IIa in cardiac hypertrophy.

### Regulation of HDAC IIb in cardiac hypertrophy

As HDAC IIa play an important role in cardiac hypertrophy, little is known about the function of the HDAC IIb (HDAC 6, 10). The mechanisms of HDAC IIb are newly discovered in recent years. As previously mentioned, HDAC6 and HDAC10 process different catalytic domain, hence the different biological function.

For HDAC6, gene knockout of Hdac5 and Hdac6 blocks the hypertrophy responses to Ang II by the COX2/PGE2 pathway. Meanwhile, sodium butyrate (NaB), an inhibitor of HDAC, inhibits COX2/PGE2 expression. Ang II can stimulate the production of ANP and phosphorylated ERK (pERK), which can also be reversed by NaB, *in vivo* and *in vitro* (70, 71). Conclusively, Ang II can trigger an HDAC5/HDAC6-dependent cardiac hypertrophy mechanism (70), which can be reversed by NaB.

HDAC10 has been identified as a polyamine deacetylase, with strong specificity for N8-acetylspermidine (72), and has not been clearly studied in cardiology. However, Hdac10 knockout in cancer cells decreases the expression of thioredoxin-interacting protein, which is a kind of endogenous thioredoxin inhibitor (73). Given the thioredoxin inhibits nuclear output of HDAC IIa (64), HDAC10 may also have the study potential in affecting genes involved in cardiac hypertrophy.

The findings suggest that HDAC II is involved in a specific pathway that the inhibits cardiac hypertrophy, but the mechanism is still unclear and further study is needed.

## Sirtuin play a protective role in cardiac hypertrophy

Class III HDACs, also known as SIRTs protein family, play a role in maintaining cardiac homeostasis. SIRT, which stands for “silent mating type information regulator,” was originally identified and named as a gene silencer that controls mating type in yeast (74). Their enzymatic activity can only be exerted in the presence of nicotinamide adenine dinucleotide (NAD +) (46), different from other HDACs. Seven sirtuin family proteins (SIRT1-7) have been identified as mammalian SIR2 orthologs, localized in different subcellular compartments. SIRT1 and 2 are in the cytoplasm, SIRT3, 4, 5 are in the mitochondria, and SIRT1, 2, 6, 7 are in the nucleus (75).

Studies show the SIRTs play a role in cardiac protection function against oxidative and aging (76, 77). The expression of SIRT1, SIRT3, and PGC-1α (Peroxisome proliferator-activated receptor gamma coactivator-1 alpha) were decreased in cardiac hypertrophy (78, 79). SIRT1 and SIRT3 enhance the deacetylation of PGC-1α, reduce oxidative stress and prevents cardiac hypertrophy (80–84). Therefore, SIRT1 and SIRT3 have protective effects on the cardiomyocytes against hypertrophy.

In addition, SIRT2 (85) and SIRT6 (86) can also prevent cardiac hypertrophy. During cardiac hypertrophy, IGF−AKT signaling pathways activates continuously. SIRT6 has been found like a negative endogenous regulator of this process in cardiomyocytes. Loss of SIRT6 resulting in H3K9 acetylation increased, and by allowing c−Jun, a stress−responsive transcription factor to interact more easily, the IGF signaling was then increased, resulting in cardiac hypertrophy (87). Moreover, nicotinamide mononucleotide adenylyltransferase is not only a key enzyme in the biosynthesis of NAD+, which related to SIRTs activation, but also inhibits angiotensin II-induced cardiac hypertrophy (88).

It is demonstrated that in muscle cells, AMP-activated protein kinase (AMPK) activation increases cellular NAD + level, increases SIRT1-mediated protein deacetylation which activates some downstream targets, such as PGC-1α and FOXO1 (Forkhead box O1). Subsequent studies have also revealed that the activation of AMPK signaling increased the transcription and protein level of NAMPT in skeletal muscles, thus stimulating Sirt1 signaling (89, 90). Interestingly, AMPK not only affects SIRTs, but also regulate the HDAC IIa. Nuclear AMPK phosphorylates residues Ser259 and Ser498 of HDAC5, and then triggers binding of HDAC5 to the signaling 14-3-3 chaperone, which is exported from the nucleus, causing histone acetylation (91–93). Meanwhile, AMPK/SNF1 (sucrose non-fermenting) pathway phosphorylate histone and activate HAT complex assembling, which triggers histone acetylation and increases transcription activity of specific genes (94–96). In this case, activation of SIRTs is associated with the AMPK/SNF1 pathway, while activation of HDAC IIa is inhibited by AMPK. This raises the intriguing possibility that the stronger AMPK activation, the higher SIRTs activation, and the more HDAC IIa are transferred to the cytoplasm, thereby contributing to cardiac hypertrophy. As mentioned above, the SIRT family has a protective effect on myocardial hypertrophy.

## Histone acetylation “reader” bromodomain protein family in cardiac hypertrophy

Histone modifications act as “marks”, and they have an “identifiers” protein family. The relationship can be described as that between books and their reader. In order to match histone acetylation, more and more “reader” proteins have been discovered and studied (97).

Bromodomain protein (BET) is an acetylated lysine binding protein. Studies on the function of BETs showed that their functional structure affects gene expression by recognizing histone acetylation and thus serves as a “reader” of epigenetic modifications, playing an important role in regulating the pathogenesis of cardiac hypertrophy (98, 99). A Small molecule JQ1, inhibits BET activation, protects pressure overload induced cardiac hypertrophy, and improves cardiac function (100).

The BET family included bromodomain-containing proteins, BRD2, BRD3, BRD4, and BRDT (101). BRD4, as a member of BETs, is generally considered to be increased in cardiac hypertrophy. Recently, acetylated histones together with BRD4 and their genomic distribution reveals their role in cardiac hypertrophy. In mice with transverse aortic constriction operation (TAC), the H3K27ac and H3K9ac genomic distribution altered 1 week after the operation and affected transcription activity (98). The loss of both histone markers in the promoter region of the gene is thought to be mediated by gene silencing and H3K27ac redistribution to the enhancer region (102). BRD4 has been shown to activate P-TEFb (positive transcription extension factor b) recruitment and transcriptional suspension release, promoting cardiac hypertrophy (99). In addition, micro-RNA (miRNA) miR-9 down-regulates the expression of BRD4 in a healthy cardiomyocyte. Hypertrophy induced stimulation significantly down-regulates miR-9, and BRD4 recruits the super enhancer gene region to initiate cardiac hypertrophy (103).

## ATP-dependent chromatin remodeling interacts with bromodomain proteins and histone deacetylases

The energy generated by ATP hydrolysis is used by ATP-dependent chromatin remodeling complexes (ADCRs) to regulate the distribution of nucleosomes and thus alter chromatin packaging state (104). This progress is also called ATP-dependent chromatin remodeling. ADCRs are generally considered to be four highly conserved families based on their ATPase domain: switching defective/sucrose non-fermenting (SWI/SNF), imitation switch (ISWS), chromodomain helicase DNA binding (CHD), and inositol requiring 80 (INO80) (105). As multiprotein complexes, ADCRs regulate the accessibility of transcription factors and appropriate gene regions on genome (105). Transcriptional regulation of these complexes has cooperation mechanisms with histone modifiers, such as HAT and HDAC (106).

BRG1 is an ATPase subunit of SWI/SNF chromatin remodeling complex, which plays a role in cardiac gene expression, as well as proliferation and differentiation of cardiomyocyte (107). The myosin is considered to be different subtypes at different developmental stages, as myosin-6 present in adult cardiomyocytes, and myosin-7 present in fetal ones. In this biological subtype conversion process, BRG1 catalyzes the HDACs and poly (ADP-ribose) polymerases (PARPs), leading to a reverse transition from myosin-7 to myosin-6 (108). Notably, in the patients with cardiomyopathy, the expression of BRG1 is increasing, which is consistent with its expression and effect in the fetal heart (109). Conclusively, BRG1 activation, together with HDAC and PARP1, indicate the subtype switch from myosin-6 to myosin-7 in pathological condition (108).

In another study, BRG1 was increased in the hypertrophy cardiomyocytes in a Dahl salt-sensitive rat model of hypertension (110), accompanied by other SWI/SNF complex subunits, such as BAF180, and BAF60C. This complex binds the *Nppa* and *Nppb*, which are considered as fetal related genes-promoter region, altering the accessibility of gene transcription region, and promoting their transcription. Conclusively, the “reader” complex, BETs, recognizes histone modifications, and activates downstream molecules or recruits related factors to further nuclear signal transduction (111).

## Crosstalk between non-coding RNA and histone acetylation

Besides interaction between histone modifications, research shows crosstalk mechanisms between histone modifications and ncRNAs, such as miRNA and long non-coding RNAs (lncRNA). For instance, *miR-449* can sponge with HDAC1, regulates the acetylation of the histones H3K4 and H3K9, thereby recruiting the transcription factor GATA4 to the cTnI (cardiac troponin I) promoter region, upregulating cTnI expression, and improving cardiac function (112). In other striated muscle, like skeletal muscle, myogenesis is promoted and regulated by *miR-1* targeting HDAC4 (113). In cardiomyocyte, we found that *pri-miR-208b* and histone-lysine N-methyltransferase EZH2 modulate gene expression in a mouse model of cardiac hypertrophy induced by pressure overload (13).

On the other hand, HDACi, such as suberanilo hydroxamic acid (SAHA) and Trichostatin A (TSA) stimulation shows the regulation of ncRNA expression in primary human endothelial cells. When the HDAC of specific genes promoter region is inhibited, EP300 will increases their histone deacetylation level, and the lncRNA regulated by EP300, such as *Malat1* (114), are activated subsequently, driven by the increased H3K4me3 at the gene promoter, and promoting the development of cardiac hypertrophy (115). Studies above reveal crosstalk mechanisms between ncRNA and histone acetylation, and show that there is still a wide range of research potential.

## Histone deacetylase as therapeutic target of cardiac hypertrophy

HDAC inhibitor (HDACi) has a wide range of cytoprotective activities, such as anti-inflammatory, antioxidant, anti-apoptotic, antifibrotic, and anti-hypertrophy, which are beneficial to the treatment of various CVDs. It has been nearly 20 years since the earliest HDACi tests. Currently, the preclinical cardiac hypertrophy models for animal have been improved, including TAC model, mouse/rat myocardial infarction models, isoproterenol or Ang II for injection or cell culture, and transgenesis mouse models. Generally, HDACi is divided into four separated classes, which are hydroxamic acids, short chain fatty acids, benzamides, and cyclic peptides. The hydroxamic acid has strong zinc-chelating properties, indicating that it has low nanomolar pan-HDAC inhibition. In contrast, the short-chain fatty acids are weak HDACi, and may be moderately selective against class I HDACs. Benzamide HDACi and the cyclic peptides are generally highly selective for HDAC1, 2, and 3. Based on previous research, preclinical studies have shown that HDACi, such as trigustatin A (TSA) and MPT0E014, can reduce cardiac remodeling and the incidence and progression of heart failure (116, 117). Main verified HDACi and their properties are listed in Table 2.

**TABLE 2:** Main HDAC inhibitors which have beneficial effect against cardiac hypertrophy.

HDAC inhibitor	Type	Target HDAC type	References
TSA	Hydroxamic acid	HDAC2, 3	(132, 145–148)
VPA	Short-chain fatty acid	HDAC1, 2, 6, 8	(145, 147, 149)
CBHA	Hydroxamic	Unclear	(150)
MGCD0103	Benzamide	Class I, IV	(126)
RGFP966	Benzamide	HDAC3	(151)
Apicidin	Cyclic peptide	HDAC1, 2, 3	(136)
NaB	Short-chain fatty acid	HDAC2, 4, 5, 6	(135)
SK-7041	Hydroxamic acid	Class I	(6)
SAHA	Hydroxamic acid	Class I, IV	(152)
Romidepsin	Cyclic peptide	Class I	(153)

CBHA, m-carboxycinnamic acid bishydroxamide; ITF2357, Givinostat; MGCD0103, mocetinostat; NaB, sodium butyrate; SAHA, suberanilo hydroxamic acid, vorinostat; TSA, trichostatin A; VPA, valproic acid.

*In vivo* animal studies have shown that 2-week treatment with the hydroxamic acid, pan-HDAC inhibitor, trichostatin A (TSA), or valproic acid can either block the development of cardiac hypertrophy in transgenic mice that overexpress an HDAC2-dependent SRF inhibitor, Hop (homeodomain-only protein) (118), no matter what strategy of cardiac hypertrophy inducing, including continuous infusion of isoproterenol or Ang II, and pressure-load model due to TAC (6, 118). More importantly, TAC-induced cardiac hypertrophy can be reversed by TSA treatment, indicating greater clinical value (6). Furthermore, valproic acid, as a weak HDAC inhibitor (119, 120), associates with a number of other pharmacological activities, including regulation of glycogen synthase kinase-3b (Gsk3β), mitogen-activated protein kinases and ion channels (121).

The effect of class I HDAC inhibition has been shown to be mainly associated with the suppression of transcriptional activity of serum response factor (SRF) and GATA4 (Table 2). In embryonic development, muscle, and neuron maintenance, SRF binds to a DNA cis element CArG box [CC(A/T)6GG], cooperates with various transcriptional factors, regulates the expression of skeletal and myocardial genes. SRF can also cooperates with GATA4, a zinc finger-containing transcription factor which highly expresses in cardiomyocytes. Both of them interact with Nkx2.5 and activate cardiac-specific gene expression (122–125). Treatment of mice overexpressing *Hdac2* with TSA increases GSK3β and following INPP5f activity, which prevents cardiac hypertrophy (51). TSA or MGCD0103, a class I and IV HDAC inhibitor, can also regulate DUSP5 mRNA expression from adverse hypertrophic stimuli, which inhibits the ERK1/2 signaling pathway. In neonatal rat ventricular myocytes (NRVMs) treated with phenylephrine, HDAC3 inhibition increased DUSP5 and inhibited ANP, *Nppb*, and *Acta1* expression after ERK1/2 nuclear phosphorylation (126). There are several other well-defined class I HDAC inhibitory pathways, including inhibiting Hop activity (118), suppressing MR (mineralocorticoid receptor) recruitment to target gene promoter (127), downregulating IGF-1/Akt pathway (128), increasing p15 and p57 (129–131), decreasing overactive autophagy (132), and upregulating miR−133a expression (133). By using these different HDACi, HDAC activity is reduced, cardiac hypertrophy related signaling pathways are suppressed, and cardiac remodeling is inhibited or even reversed. However, some unknown mechanisms remain to be solved (17).

Class II HDACi are also defined to protect against cardiac hypertrophy by several pathways, including reversing caspase-3 and HDAC4 levels (134), increasing cardiac superoxide dismutase and micro-vessel genesis to attenuate cardiac hypertrophy (135), increasing p38 phosphorylation (134, 135), blocking the increases in heart weight and the level of several mRNAs, such as ANP, BNP, β-MHC, and IL-1 induced by β-angiotensin II, indicating a myocadial hypertrophy status (70). However, it should be noted that the HDAC II inhibitor, NaB, is also a short-chain fatty acid like valproic acid, which implies low specificity and may have many other pharmacological activities.

## Conclusion

In the last 20 years, there have been significant advances in understanding the function and regulatory patterns of HDACs in the physiological or pathological heart, as well as a clearer understanding of histone acetylation/deacetylation. Further discovery of the mechanisms by which HDAC interacts with histone acetylation/deacetylation cofactors will bring new insights into the understanding of this complex biological process and contribute to a variety of heart diseases, particularly cardiac hypertrophy.

Here, we review the mechanisms of histone acetylation/deacetylation in cardiac hypertrophy and briefly introduce therapeutic HDAC inhibition (Figure 1). This article reviews the specific mechanism of HDAC regulating cardiac hypertrophy. HDAC I catalyzes the histone lysine residue, induces chromatin condensation, and reduces the transcription activity of anti-hypertrophy genes. Furthermore, HDAC IIa works differently. HDAC IIa is considered to be weak catalyze activity. However, by binding to transcript factors such as MEF2 and GATA4, HDAC represses the recruitment of HAT, which will acetylate the lysine residue, leading to increased gene expression. Besides, multiple types of HDACi may affect HDAC I and IIa. Meanwhile, HDAC III, which is also regarded as SIRTs, is involved in reducing oxidative stress and preventing cardiac hypertrophy. In addition, the BET family acts as a “reader” of histone acetylation, recognizes the acetylated lysine histones, and activates the promoter region of cardiac hypertrophy genes. The BET family also participates in ADCR-mediated chromatin remodeling process, and affects the transcriptional activity of specific genes. Last but not the least, there are multiple crosstalk mechanisms among HDAC and other epigenetic modifications. Their interactions constitute a complicated regulatory network among of histone modifications, and affect cardiac hypertrophy through different pathways.

**FIGURE 2:**
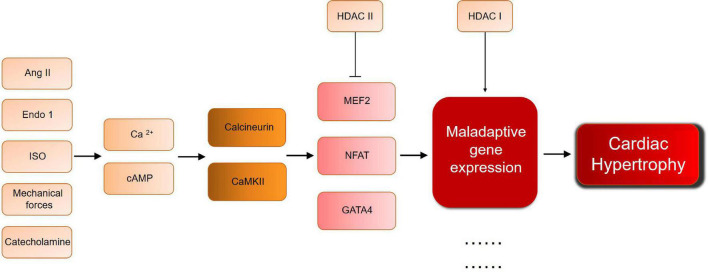
A brief scheme of cardiac hypertrophy development and the key molecules mentioned in this review. Ang II, angiotensin II; Ca, calcium; CaMKII, calcium/calmodulin dependent protein kinase; Endo 1, Endothelin 1; GATA, GATA binding protein; HDAC, histone deacetylase; ISO, isoproterenol; MEF, myocyte-specific enhancer factor; NFAT, nuclear factor of activated T cells.

Although histone modification has been proved to play an important role in cardiac hypertrophy, its use as an explanation for cardiac hypertrophy remains limited. For example, the inability of a single study to link all mechanisms solves the problem of understanding the complex mechanisms underlying the development of cardiac hypertrophy. In addition, most studies focus on model animals with cardiac hypertrophy stimulated by surgical intervention or gene knockout. However, the human cardiac hypertrophy is a long-term, chronic process, which is affected by environment, psychology, society, and other variables. Simple animal models do not fully mimic the real pathogenesis and the course of disease. Furthermore, HDAC regulating mechanisms are still in the downstream of cardiac hypertrophy development. As Figure 2 shows, different pathological condition will both go through calcium/cAMP changes, and lead to calcuneurin or CaMKII pathway. In the downstream, MEF/NFAT/GATA4 activate and interact with HDACs, no matter what the pathological condition is, which means HDAC will be a critical therapeutic target, for the general effect of promoting/inhibiting hypertrophy. But for the upstream mechanisms of cardiac hypertrophy, it still remains problems to be solved. There are too many complicated and undetected stimulation, and their relationship with HDAC is still worthy to be studied, especially those who can directly affect HDAC activation. For example, the AMPK regulation between SIRTs and HDAC IIa. Moreover, effective selective HDACi are usually not organ-specific. As a normal biological process, histone acetylation/deacetylation should not be simply and globally suppressed, hence the need of developing more specific, reversible inhibitors, and more safety assessment and clinical trial before clinical application. This requires more exploration and research into the mechanism itself. Thus, further efforts are required to study the mechanisms.

In this article, we present currently known knowledge about the key enzyme family, HDAC, as far as possible. As an enzyme family, their participation in histone deacetylation, the relationship between HDAC and other transcription factors, and the connection with other histone modifications are analyzed. Meanwhile, we introduce more partner molecules, such as p300, MEF, GATA, BETs, ADCRs, and ncRNAs, are also participant in epigenetic modification. Furthermore, we recognize that the revealed mechanisms and current studies still require more efforts to dig deeper in the application of HDACi. However, efforts to further study HDAC are still recommended, and more and more studies are revealing histone modification mechanisms. It will provide a theoretical support for HDAC as a therapeutic target for cardiac hypertrophy in the near future.

## Author contributions

YH and JN carried out investigations. YH and LN wrote the manuscript. LN and DW supervised the entire study, revised the manuscript, and provided the funding. All authors contributed to the writing and editing of the manuscript.
